# Microspore embryogenesis induction by mannitol and TSA results in a complex regulation of epigenetic dynamics and gene expression in bread wheat

**DOI:** 10.3389/fpls.2022.1058421

**Published:** 2023-01-09

**Authors:** Isabel Valero-Rubira, Ana María Castillo, María Ángela Burrell, Maria Pilar Vallés

**Affiliations:** ^1^ Departamento de Genética y Producción Vegetal, Estación Experimental de Aula Dei, Consejo Superior de Investigaciones Científicas (EEAD-CSIC), Zaragoza, Spain; ^2^ Departamento de Patología, Anatomía y Fisiología, Facultad de Ciencias, Universidad de Navarra, Pamplona, Spain

**Keywords:** bread wheat, microspore embryogenesis, mannitol, trichostatin A, histones, epigenetics, gene expression

## Abstract

Reprogramming of microspores development towards embryogenesis mediated by stress treatment constitutes the basis of doubled haploid production. Recently, compounds that alter histone post-translational modifications (PTMs) have been reported to enhance microspore embryogenesis (ME), by altering histones acetylation or methylation. However, epigenetic mechanisms underlying ME induction efficiency are poorly understood. In this study, the epigenetic dynamics and the expression of genes associated with histone PTMs and ME induction were studied in two bread wheat cultivars with different ME response. Microspores isolated at 0, 3 and 5 days, treated with 0.7M mannitol (MAN) and 0.7M mannitol plus 0.4µM trichostatin A (TSA), which induced ME more efficiently, were analyzed. An additional control of gametophytic development was included. Microspores epigenetic state at the onset of ME induction was distinctive between cultivars by the ratio of H3 variants and their acetylated forms, the localization and percentage of labeled microspores with H3K9ac, H4K5ac, H4K16ac, H3K9me2 and H3K27me3, and the expression of genes related to pollen development. These results indicated that microspores of the high responding cultivar could be at a less advanced stage in pollen development. MAN and TSA resulted in a hyperacetylation of H3.2, with a greater effect of TSA. Histone PTMs were differentially affected by both treatments, with acetylation being most concerned. The effect of TSA was observed in the H4K5ac localization pattern at 3dT in the mid-low responding cultivar. Three gene networks linked to ME response were identified. *TaHDT1, TaHAG2, TaYAO*, *TaNFD6-A*, *TabZIPF1* and *TaAGO802-B*, associated with pollen development, were down-regulated. *TaHDA15*, *TaHAG3*, *TaHAM, TaYUC11D*, *Ta-2B-LBD16 TaMS1* and *TaDRM3* constituted a network implicated in morphological changes by auxin signaling and cell wall modification up-regulated at 3dT. The last network included *TaHDA18, TaHAC1*, *TaHAC4, TaABI5*, *TaATG18fD, TaSDG1a-7A* and was related to ABA and ethylene hormone signaling pathways, DNA methylation and autophagy processes, reaching the highest expression at 5dT. The results indicated that TSA mainly modified the regulation of genes related to pollen and auxin signaling. This study represents a breakthrough in identifying the epigenetic dynamics and the molecular mechanisms governing ME induction efficiency, with relevance to recalcitrant wheat genotypes and other crops.

## Introduction

Microspore embryogenesis (ME) constitutes a process in which the microspore changes its developmental pathway from a gametophytic towards a sporophytic. This change is triggered by a stress treatment, followed by an *in vitro* culture phase in which embryos and haploid plants are finally formed (Soriano et al., 2013). Doubled haploid (DH) plants can be obtained spontaneously by nuclear fusion or endoreduplication, during the first microspore divisions, or can be induced by the action of chromosome doubling agents (González-Melendi et al., 2005; Griggs and Zheng, 2016). DH plants have a great value to produce new varieties or inbred lines because complete homozygosis is achieved in one generation.

In recent years, increasing evidence has been accumulated on how the dynamics of epigenetic processes regulates gene expression and establishes genomic elements in adaptive responses to developmental transitions. Epigenetic modifications have been reported to play an important role in reproductive development, abiotic stress response and plant regeneration, processes which are involved in ME (Begcy and Dresselhaus, 2018; Lee and Seo, 2018; Mozgova et al., 2019; Ono and Kinoshita, 2021).

Changes in histone post-translational modifications (PTMs), DNA methylation as well as regulation of small RNA and long noncoding RNA pathways have been described as the major epigenetic mechanisms in plants (Pikaard and Scheid, 2014). PTMs of histone-tails can alter the interaction between histones and DNA directly or by recruiting specific effectors such as transcriptional regulators or chromatin remodelers (for review see Tessarz and Kouzarides, 2014). Acetylation and methylation are the two most common histone PTMs involved in activation and repression of transcription, respectively. Histone deacetylases (HDAC) and acetyltransferases (HAT) regulate the balance of histone acetylation by modifying lysine residues, leading to a chromatin compaction/decompaction that facilitates the repression/activation of gene transcription (for review see Bannister and Kouzarides, 2011). Histone lysine residues can be mono-, di- or tri-methylated in a dynamic way by the action of histone lysine methyltransferases (HKMT) and histone demethylases (for review see Bannister and Kouzarides, 2011).

Histone hyperacetylation and DNA hypomethylation have been directly associated to ME induction by the application of a heat stress treatment in the model species *Brassica napus* (Solis et al., 2012; Li et al., 2014a). Furthermore, the application of inhibitors of enzymes involved in the modification of epigenetic marks has been shown to be a successful strategy to increase the efficiency of ME (for review see Testillano, 2019). Thus, trichostatin A (TSA), a HDAC inhibitor acting over acetylation of histones and other proteins related to cytoskeleton, increased the efficiency of microspore reprogramming in *Brassica* (Li et al., 2014a; Zhang et al., 2016) and barley (Pandey et al., 2017). Studies performed in barley indicated that there was a very intricate interaction between the histone acetylation and methylation after TSA application (Pandey et al., 2017). Other histone deacetylases inhibitors such as suberoylanilide hydroxamic acid (SAHA) and sodium butyrate have also been used to enhance ME in *Brassica* (Zhang et al., 2016). Also, the application of 5-azacytidine, which causes a global DNA demethylation, or BIX, a histone lysine methyltransferase inhibitor, promotes ME in *Brassica*, barley and triticale (Solis et al., 2015; Berenguer et al., 2017; Nowicka et al., 2019).

Different approaches have been described for the application of TSA to increase ME efficiency in wheat. Thus, green plant regeneration was enhanced in different cultivars applying TSA in the microspore isolation procedure or in the culture medium, after a cold stress treatment (Jiang et al., 2017; Wang et al., 2019). However, a comparison of different strategies showed that the application of 0.4 µM TSA simultaneously with 0.7 M mannitol for 5 days was the most efficient for DH production, increasing twice and four times the number of green plants compared to mannitol in a high and a mid-low responding bread wheat cultivars, respectively (Castillo et al., 2020).

Although different protocols are available for ME in wheat (Castillo et al., 2021; Jensen et al., 2022), their efficiency depends largely on the genotype (Lantos et al., 2013; Weigt et al., 2019; Castillo et al., 2019). The genetic control of ME is quite complex (Muñoz-Amatriaín et al., 2008; Nielsen et al., 2015; Abd ElFath et al., 2020). Besides the genetic control, the epigenetic state of the microspores before ME induction could also play and important role. Thus, microspores from a high ME responding line of triticale had a higher level of DNA methylation than a low responding one (Nowicka et al., 2019). Different levels of endogenous ABA as well as gene transcripts related to ME have been also described in genotypes with different ME response in *Brassica*, wheat and triticale (Dubas et al., 2013; Sánchez-Díaz et al., 2013; Nowicka et al., 2019).

Little is known about the dynamics of histone PTMs and their impact on transcriptional regulation of wheat ME. A recent study applying TSA after a cold treatment shows that TSA up-regulates genes involved in cell division, DNA organization, signaling cascades, hormone mediated signaling, and cell wall and cytoskeleton modifications (Jensen, 2021). To provide new insights into wheat ME induction mechanisms, the dynamics of acetylation and methylation histone PTMs was analyzed with a mannitol and a mannitol plus TSA treatment, which induced ME more efficiently, in two bread wheat cultivars with different ME response. The expression of genes involved in PTMs and key genes for ME and their correlations were also studied. For the first time, the epigenetic dynamics and gene regulation are discussed in wheat ME induction depending on cultivars and treatments.

## Material and methods

### Material, growing conditions of the donor plants and harvest of the spikes

The spring cultivars (cvs) of bread wheat Pavon and Caramba, which have a high and a mid-low ME response, respectively, were used in this study. Growth conditions of the mother plants and harvest and sterilization of spikes were performed as described by Castillo et al. (2021). Anthers containing most microspores at the mid to late uninucleated stage, determined by DAPI (4´, 6-Diamidine-2’-phenylindole dihydrochloride) staining were excised and brought under stress treatments. For selection of the spikes at this stage of development, tillers with 3-5 cm length from the distal part of the spike to the basal part of the flag leaf were harvested. Some spikes were harvested two days later to allow microspores to progress into gametophytic development (GD).

### Application of TSA in combination with a mannitol stress treatment

Comparison of different TSA application strategies showed that the incorporation of TSA in a mannitol treatment was the best to enhance wheat ME (Castillo et al., 2020). Therefore, the effect of 0.4 µM TSA (Sigma T8852) in combination with 0.7 M mannitol stress treatment was assayed. TSA was dissolved in 1% DMSO (final concentration in the medium). Freshly excised anthers were randomly distributed in 6 cm Ø Petri dishes containing solidified 0.7 M mannitol stress medium (SM medium, Castillo et al., 2021) (MAN) or in mannitol plus 0.4 µM TSA (TSA) (Castillo et al., 2020) and placed in the dark at 25°C. Microspores from anthers after 3 and 5 days of MAN (3dT-MAN, 5dT-MAN) and TSA (3dT-TSA, 5dT-TSA) treatments from Pavon and Caramba were isolated following the protocol described by Castillo et al. (2000). Isolated microspores from freshly harvested anthers (0dT) were used as control. In addition, isolated microspores at GD were used as a second control to discriminate the gametophytic and the sporophytic pathways.

### Protein Analysis by Western blot

Isolated microspores of Pavon and Caramba from 0dT, GD, 3dT-MAN, 5dT-MAN, 3dT-TSA, and 5dT-TSA were used. Leaves from 30 day-old plants, young embryos (14 days after pollination) and pollen grains were also included in this analysis. Proteins were extracted using the buffer containing 150 mM NaCl, 2 mM EDTA and 50 mM NaH_2_PO_4_, 2% SDS, pH 6. After homogenization, samples were boiled (95°C 10 min) and centrifuged twice at 15000 g, 10 min at 4°C. Protein extracts were quantified using Qubit Protein BR Assay kit following manufacturer’s instructions in fluorimeter Qubit4. Ten µg of total proteins from all samples, except from embryos (5 µg), were electrophoresed on a Mini-PROTEAN TGX 12% SDS-PAGE gel under reducing conditions. Proteins were transferred to a polyvinylidene difluoride membrane and blocked with 5% BSA in TBS-T (20 mM Tris, 150 mM NaCl, pH 7.5, and 0.1% Tween 20). The blots were incubated for 2 h with anti-histone H3 (clone A3S, Millipore), anti-histone H4 (clone 62-141-13, Millipore), anti-pan-acetyl-histone H3 (K9, K14, K18, K23, K27) (ab47915, Abcam) or anti-pan-acetyl-histone H4 (K5, K8, K12, K16) (PA1-84526 Invitrogen), all of them at 1:1000 dilution. Secondary goat anti-rabbit IgG peroxidase conjugated antibody (AP132P, Millipore) was used at a 1:1000 dilution and signals were detected using enhanced chemiluminescence (Clarity Western ECL substrate, BIORAD). Western blot was performed in two independent biological replicates.

### Immunofluorescence analysis

Isolated microspores of both cultivars from 0dT, GD, 3dT-MAN, 5dT-MAN, 3dT-TSA, and 5dT-TSA were fixed in 4% paraformaldehyde and 2% sucrose in PBS, pH 7.3, overnight at 4°C. After three washes in PBS for 10 min, microspores were pre-embedded in 2% agarose and post-fixed in the same fixative solution during 2 h at 4°C. The samples were washed twice in PBS before being dehydrated in an ethanol series (30, 50 and 70%) at 4°C, processed in an automatic Vacuum Infiltration Processor (Sakura 4893-Floor E150, BAYER, Barcelona, Spain), and embedded in paraffin blocks. Four µm-thick sections were cut using a rotary microtome (Microm HM 340E, Bio Optica, Milan, Italy) with steel blades, and were collected on special slides (Menzel-Glaser, J1800AMNN, Braunschweig, Germany) to facilitate their adherence and avoid their loss during the performance of the immunohistochemical techniques.

The sections were deparaffinized with xylene and rehydrated with graded ethanol to water. Before the incubation with the primary antibodies, samples were treated with 0.05 M NH_4_Cl and 0.05 M NaBH_4_ in TBS (0.05 M TRIS, 0.5 M NaCl, pH 7.36) to partially reduce the autofluorescence of aldehydes (highly abundant in the exine of microspores) and were blocked with a 2% BSA solution at RT. The following primary antibodies, raised in rabbit, were used: anti-histone H3K9ac (EMD Millipore, Cat. No. 06-942; dilution 1:100), anti-histone H4K5ac (EMD Millipore, Cat. No. 07-327; dilution 1:100), anti-histone H4K16ac (EMD Millipore, Cat. No. 07-329; dilution 1:100), anti-histone H3K9me2 (EMD Millipore, Cat. No. 07-441; dilution 1:100) and anti-histone H3K27me3 (EMD Millipore, Cat. No. 07-449; dilution 1:50). The samples were incubated with primary antibodies overnight at 4°C. Goat anti-rabbit Alexa Fluor 488 (Sigma, Cat. No. SAB4600044; dilution 1:100) was applied as secondary antibody during 30 min at RT. Fluoromount G with DAPI (Invitrogen, Cat. No. 00-4959-52) was used as an aqueous mounting medium.

To study the localization pattern of epigenetic marks and to quantify the number of labeled microspores and nuclei, an inverted microscope Nikon Eclipse-T300 was used and fluorochrome Alexa Fluor 488 was excited at 460 nm, and emission was detected with a 505-520 nm long-pass filter. Microspores nuclei were counterstained with DAPI, excited at 350 nm in combination with a 400-420 nm long-pass filter. Images were recorded by the Digital sight DS-5MC camera with NIS-D software. Immunohistochemical analysis was performed in two independent biological replicates, containing an average of five technical replicates for each mark (H3K9ac, H4K5ac, H4K16ac, H3K9me2 and H3K27me3). An average of 1500 and 1000 microspores and 400 and 250 nuclei from Pavon and Caramba were analyzed, respectively.

### Identification of selected genes in the wheat genome

Genes associated with ME induction response were selected based on a previous study performed in barley (Sánchez-Díaz, 2014). In this study, an Affymetrix Barley1 GeneChip microarray analysis was carried out on anthers before stress treatment, after 4 days of 0.7 M mannitol treatment, and after 4 and 8 days of culture, following the procedures described in Muñoz-Amatriaín et al. (2006). Identification and clustering of the differentially expressed genes allowed the selection of candidate genes that characterized barley ME induction. Among them, 20 barley probe set related to HDACs, HATs, and major processes involved in ME induction were selected (**Supplementary Table 1**).

Wheat genes with homology to barley probe set were identified based on sequence homology by BLASTN of barley sequences exported from HarvEST : Barley version 2.26 (http://www.harvest.ucr.edu), against *Triticum aestivum* IWGSC (Genomic sequence) on Ensembl Plants, which hosts the RefSeq v1.0 assembly from the IWGSC (http://plants.ensembl.org/Triticum_aestivum/Tools/Blast) or against WheatExp: An Expression Database for Polyploid Wheat (https://wheat.pw.usda.gov/WheatExp/). Overlapping genes with higher scores were selected, and homologues genes in bread wheat and orthologues in *Arabidopsis thaliana* and *Oryza sativa* Japonica Group were identified on Ensembl Plants. Only *TaMS1* gene was directly selected from literature (**Supplementary Table 1**).

If primers were not available in the literature, the cDNA sequence of genes based on Ensembl Plants/*Triticum_aestivum* was used for designing specific primer pairs by Primer-BLAST (NCBI), which uses Primer3 (Ye et al., 2012) (**Supplementary Table 2**).

Selected genes ontology analyses were performed based on RefSeq v1.0 assembly by AgriGO v2.0, a database (Tian et al., 2017) (**Supplementary Table 3**).

### Transcript level analysis by quantitative RT-PCR

Total RNA of three independent biological replicates from 0dT, GD, 3dT-MAN, 5dT-MAN, 3dT-TSA, and 5dT-TSA from Pavon and Caramba was isolated using TRIzol Reagent (Ambion, Life technologies) and passed through RNeasy columns (Qiagen) for further clean up. Double-stranded cDNA was synthesized using the M-MLV Reverse Transcriptase kit from Promega. qRT-PCR reactions were performed with PowerUp SYBR Green Master Mix (Applied Biosystems) and ROX (ROX Reference Dye, Invitrogen). The reaction conditions were optimized at: 95°C for 10 min, followed by 40 cycles of 95°C for 15 s and 60°C for 1 min, in the QuantStudio3 System (Applied Biosystems), using the *Ta.27771* and *Ta.2291* as reference genes (Paolacci et al., 2009). Two technical replicates were performed for each biological replicate in each gene.

### Gene expression pattern in different stresses and tissues in a wheat *in silico* analysis

The expVIP Wheat Expression Browser (http://www.wheat-expression.com) was used to study the expression of selected genes in different studies (Ramírez-González et al., 2018). The relative transcript abundance (per 10 million reads) from each gene obtained from the databases was presented as a heat map (Graphad Prism 7).

### Statistical analysis

A densitometry analysis was performed on images of western blotting membranes, obtained in the ChemiDoc Imaging System (BIO-RAD), using the gel analyzer tool from Image-J software. Densitometry data allowed the estimation of the H3.1/H3.2, H3.1ac/H3.1, H3.2ac/H3.2 and H4ac/H4 ratios. In the immunofluorescence analysis, the following variables were calculated: the percentage of labeled microspores, that is, the number of microspores with each epigenetic marks over the total number of microspores, and the percentage of labeled nuclei, that is, the number of microspores with each epigenetic marks in the nuclei over the total number of microspores stained with DAPI. Normality and homogeneity of variance were tested using the Shapiro-Wilk and Levene´s tests, respectively. Data from the qRT-PCR reactions were analyzed using the Livak (2-∆∆CT) calculation method (Livak and Schmittgen, 2001). For the calculation of the standard errors, the values of the relative expression level (2-∆∆CT) were used. For statistical analysis, the values of fold change (2 -∆∆CT) were transformed with log2 to correct the heterogeneity of variance. A one-way analysis of variance (ANOVA) was used, and significant differences among treatments were determined by the Duncan test (p ≤0.05). Statistical analysis of these variables was performed using IBM SPSS statistics version 27.0.1. (IBM Corp, 2020).

All possible pairwise correlations of the expression of the studied genes over samples were calculated using Pearson´s coefficient. A principal component analysis (PCA) was performed to analyze the association between relative gene expression and the course of ME inducing treatments, using the program R 4.1.3 (R Core Team, 2021).

## Results

### Histone H3 and H4 acetylation dynamics in microspore embryogenesis induction

**Figure 1:**
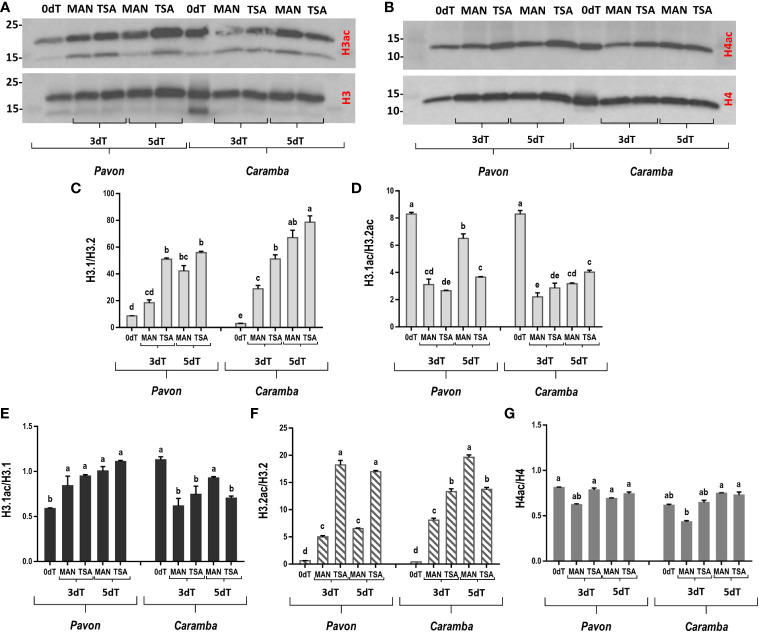
Effect of MAN and TSA treatments on histones H3 and H4 acetylation. **(A, B)** Immunoblot of histones H3 and H4 and pan-acetylated H3 and H4 (H3ac and H4ac, respectively). **(C, D)** Quantitative analysis of the H3.1/H3.2 and H3.1ac/H3.2ac ratios, respectively. **(E, F)** Quantitative analysis of the H3.1 and H3.2 acetylation ratio **(G)** Quantitative analysis of the H4 acetylation ratio. Microspores before stress treatment (0dT), after 3 and 5 days with mannitol treatment (3dT-MAN, 5dT-MAN), and after 3 and 5 days with mannitol plus TSA treatment (3dT-TSA, 5dT-TSA) from Pavon (on the left) and Caramba (on the right). Bars represent the standard errors of the means. Histone H3.1/H3.2, H3.1ac/H3.2ac, H3.1ac/H3.1, H3.2ac/H3.2 and H4ac/H4 ratios with the same letter within each ratio are not significantly different (P˂0.05) according to the Duncan test.

To determine if MAN and TSA treatments modified microspores global histone acetylation, a western blot assay was performed using a pan-acetylated Lys antibody for histones H3 and H4 (**Figures 1A, B**). Antibodies against unmodified histone H3 and H4 were used as controls. In the text, only statistically significant differences were described. However, in some cases a tendency to statistical significance has been described as slight.

Two histone H3 variants with different molecular weight (17 KDa and 14 KDa, approximately) were detected in both cultivars (**Figure 1A**). These proteins corresponded to the major H3.1 variant and the minor H3.2 variant (H3.3-type, Waterborg, 1991). Quantitative analysis of the H3.1/H3.2 ratio showed a low value at 0dT, which increased greatly with MAN and TSA treatments, reaching the highest value at 5dT (**Figure 1C**). This ratio was higher with TSA than with MAN in both cultivars at 3dT. Given the change in the H3.1/H3.2 ratio during ME induction, the study was extended to young zygotic embryos, leaves, and GD, including mature pollen grains (**Supplementary Figure 1A**). The two variants were observed in all samples, although there were differences between cultivars, particularly in leaves (**Supplementary Figures 1A, B**). It was noteworthy the increase of H3.1/H3.2 in mature pollen (P) in both cultivars (**Supplementary Figure 1B**). Embryos showed the highest and the lowest amount of H3.1 and H3.2 variants, respectively, but quantification of H3.1/H3.2 was not possible.

Acetylation of both H3.1 and H3.2 variants was observed in all microspore samples (**Figure 1A**). The highest H3.1ac/H3.2ac ratio was showed at 0dT, and treatments reduced this ratio in both cultivars, especially at 3dT (**Figure 1D**). However, it increased greatly at 5dT-MAN in Pavon. Acetylation ratio of each variant was also studied (**Figures 1E, F**). At 0dT, H3.1ac/H3.1 was twice higher in Caramba than in Pavon. MAN and TSA treatments increased this ratio in Pavon but decreased it in Caramba (**Figure 1E**). H3.2ac/H3.2 had low and similar values at 0dT, but MAN and TSA induced H3.2 acetylation in both cultivars (**Figure 1F**). However, TSA enhanced acetylation up to approximately 4-fold over MAN at 3dT and 5dT in Pavon, but 1.5-fold only at 3dT in Caramba.

Analysis with H4 antibody showed that it was acetylated in all samples at a similar ratio to those of H3.1 variant (**Figures 1B, E, G**). It should be noted that MAN caused a slight decrease in H4 acetylation at 3dT in both cultivars, followed by an increase at 5dT in Caramba, reaching similar values in both cultivars (**Figure 1G**). The acetylation ratio at 3dT-TSA was slightly higher than at 3dT-MAN, but similar values were observed at 5dT.

### Immunolocalization and dynamics of epigenetic marks in wheat microspores

Epigenetic dynamics were characterized by the pattern of intracellular localization and the percentage of labeled microspores or nuclei with specific antibodies for histone acetylation (H3K9ac, H4K5ac, H4K16ac) and methylation (H3K9me2 and H3K27me3). The percentage of labeled nuclei was only noted if it was different from that of the labeled microspores.

**Figure 2:**
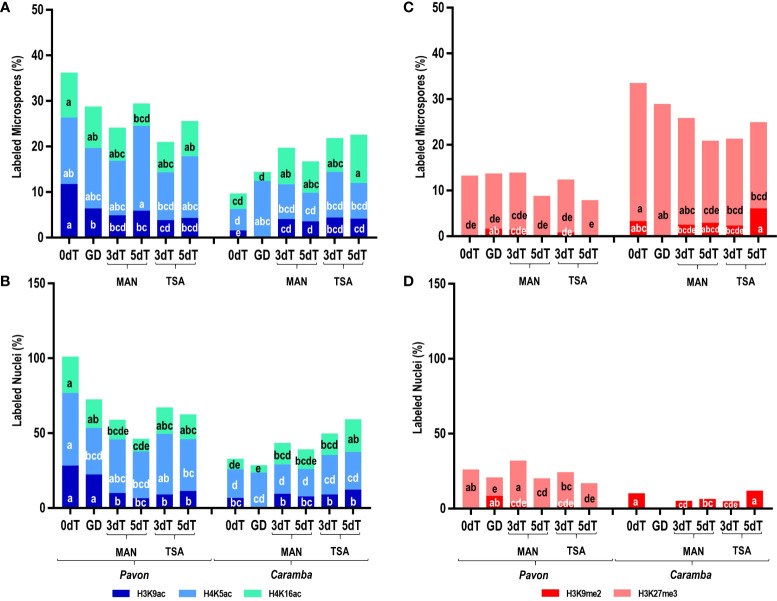
Effect of MAN and TSA on histone PTMs. **(A, B)** Percentage of labeled microspores and nuclei, respectively, with the acetylation marks H3K9ac (dark blue), H4K5ac (light blue), H4K16ac (green). **(C, D)** Percentage of labeled microspores and nuclei, respectively, with the methylation marks H3K9me2 (red), H3K27me3 (pink). Microspores before stress treatment (0dT), microspores under gametophyte development (GD), microspores after 3 and 5 days with mannitol treatment (3dT-MAN, 5dT-MAN) and after 3 and 5 days with mannitol plus TSA treatment (3dT-TSA, 5dT-TSA) from Pavon (on the left) and Caramba (on the right). Percentages of labeled microspore and nuclei with the same letter within each epigenetic mark are not significantly different (P˂0.05) according to the Duncan test.

**Figure 3:**
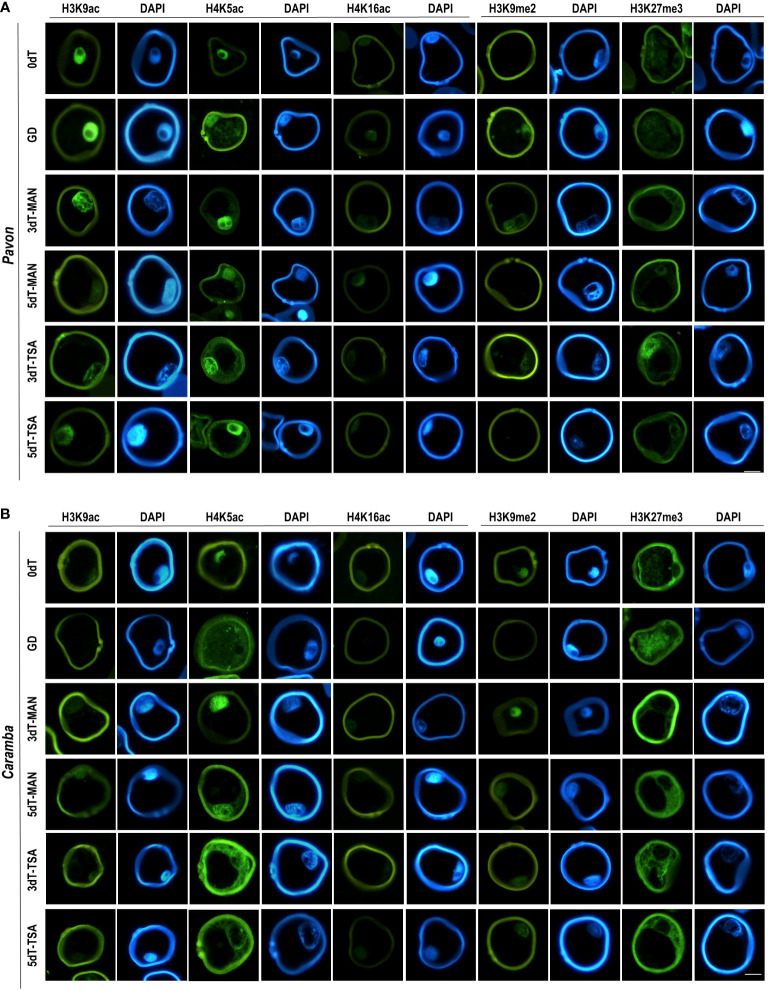
Immunolocalization of histone PTMs in microspores before stress treatment (0dT), microspores under gametophytic development (GD), microspores after 3 and 5 days with mannitol treatment (3dT-MAN, 5dT-MAN) and after 3 and 5 days with mannitol plus TSA treatment (3dT-TSA, 5dT-TSA) from Pavon **(A)** and Caramba **(B)**. H3K9ac, H4K5ac, H4K16ac, H3K9me2 and H3K27me3 antibodies labeling in green, DAPI in blue. Scale bar = 20 µm.

Uninucleate microspores from the two cultivars at 0dT showed large differences in epigenetic state, although they were at a similar morphological stage of development according to microscopic visualization. The sum of the percentages of microspores with acetylation marks was 35.85% in Pavon but only 9.29% in Caramba, with the values for each mark being lower in Caramba (**Figure 2A**). In contrast to acetylation, the sum of the percentages of microspores with methylation marks was lower in Pavon (12.89%) than in Caramba (33.14%) (**Figure 2C**). It should be noted the low number of microspores with H3K27me3 and the absence of H3K9me2 mark in Pavon. Furthermore, all acetylation marks were exclusively located in the nucleus in both cultivars (**Figures 3A, B**). H3K27me3 was observed in the cytoplasm (including the vacuole) in both cultivars and only in the nucleus in Pavon (**Figures 3A, B**).

Differences in the dynamics of PTMs at GD were also observed between cultivars. The sum of percentages of acetylation decreased in Pavon as compared with 0dT (28.39% vs 35.85%), mainly due to the lower value of H3K9ac (6.03% vs 11.40%) (**Figure 2A**). There was a global increase of acetylation in Caramba (14.01%) due to H4K5ac (12.07%), which reached similar percentages to Pavon (13.25%). H4K5ac mark was observed in the nucleus and the cytoplasm (including the vacuole) in both cultivars and H3K9ac was absent in Caramba (**Figures 3A, B**). Concerning methylation, H3K9me2 was detected in a low percentage of nuclei (7.31%) in Pavon and was absent in Caramba (**Figure 2D**), and H3K27me3 was localized in the cytoplasm (including the vacuole) in both cultivars (**Figure 3B**).

Induction treatment with MAN modified the epigenetic state of microspores compared to 0dT and GD, and determined differences between cultivars. In Pavon, the sum of the percentages of acetylated microspores at 3dT (23.76%) decreased as compared to 0dT (35.85%) or GD (28.39%), mainly due to lower value of H3K9ac (4.51% vs 11.40%) (**Figure 2A**). A decrease was also observed in the percentage of labeled nuclei with H4K16ac (**Figure 2B**). However, the percentage of acetylated microspores rose at 5dT-MAN (29.07%), mainly due to the H4K5ac mark, although this percentage decreased in the nuclei (**Figure 2B**). In Caramba, the dynamics of epigenetic marks was different from that of Pavon. An increase in the percentage of acetylated microspores was observed at 3dT with respect to 0dT (19.36% vs 9.29%), being significant for H3K9ac (3.67% vs 1.19%) and H4K16ac (8.06% vs 3.42%) (**Figure 2A**). Nevertheless, the differences were not significant in the nuclei (**Figure 2B**). A slight decrease in all acetylation marks was noticed at 5dT (**Figure 2A**).

Regarding methylation, the percentage of labeled microspores with H3K9me2 was very low at 3dT-MAN (1.08%), and it was absent at 5dT-MAN in Pavon (**Figure 2C**). In Caramba, this percentage was not modified with MAN, but the percentage of labeled nuclei decreased (**Figure 2D**). Furthermore, the percentage of microspores with H3K27me3 decreased at 5dT (17.98%) compared to 0dT (30.18%).

The localization of H4K5ac and H3K27me3 with MAN also revealed differences between the two cultivars. H4K5ac was detected in the nucleus and cytoplasm at both 3dT and 5dT in Pavon (**Figure 3A**). In Caramba, it was only observed in the nucleus at 3dT, but also in the cytoplasm at 5dT (**Figure 3B**). H3K27me3 was present in the nucleus and cytoplasm in Pavon (**Figure 3A**), but only in the cytoplasm in Caramba (**Figure 3B**).

TSA treatment produced modifications in the epigenetic state of the microspores compared to MAN. TSA decreased the percentages of microspores with acetylation marks in Pavon (20.61% at 3dT-TSA and 25.25% at 5dT-TSA vs 23.76% at 3dT-MAN and 29.07% at 5dT-MAN) (**Figure 2A**). However, the sum of the percentages of labeled nuclei was higher at 5dT-TSA than at 5dT-MAN, mainly due to the H4K16ac mark (**Figure 2B**). In Caramba, higher percentages of acetylated microspores were observed with TSA than with MAN, especially at 5dT (22.24% vs 16.37%), highlighting the increase of the H4K16ac mark. The most remarkable change produced by TSA was the localization of H4K5ac in both the nucleus and cytoplasm at 3dT-TSA in Caramba (**Figure 3B**). Finally, TSA resulted in an increase in the percentage of labeled nuclei with H3K9me2 at 5dT-TSA compared to 5dT-MAN in Caramba, and a decrease in the percentage of nuclei with H3K27me3 at 3dT-TSA in Pavon (**Figure 2D**).

### Transcriptional changes associated to microspore embryogenesis induction by MAN and TSA treatments

The immunohistochemical analysis revealed that MAN and TSA modified the epigenetic state of microspores. Therefore, the dynamics of transcriptional changes in genes controlling histone acetylation state and genes involved in key processes of ME induction produced by treatments was studied. The candidate genes were selected based on a previous study on barley ME induction (Sánchez-Díaz, 2014). Genes were associated with GO terms as a source of information about gene functions (**Supplementary Table 3**).

#### Expression of histone deacetylases and acetyltransferases genes

Histone acetylation depends on the balance between HDAC and HAT. In this study, six wheat HDAC genes (*TaHDA8*, *TaHDA15*, *TaHDA18*, *TaHDT1*, *TaHDT2* and *TaTAF14*) and five HAT genes (*TaHAG2*, *TaHAG3*, *TaHAM*, *TaHAC1* and *TaHAC4*) were studied. Three HDAC genes were not considered for further analysis as *TaHDA8* was not amplified, and *TaHDT2* and *TaTAF14* showed no differences between the samples.

**Figure 4:**
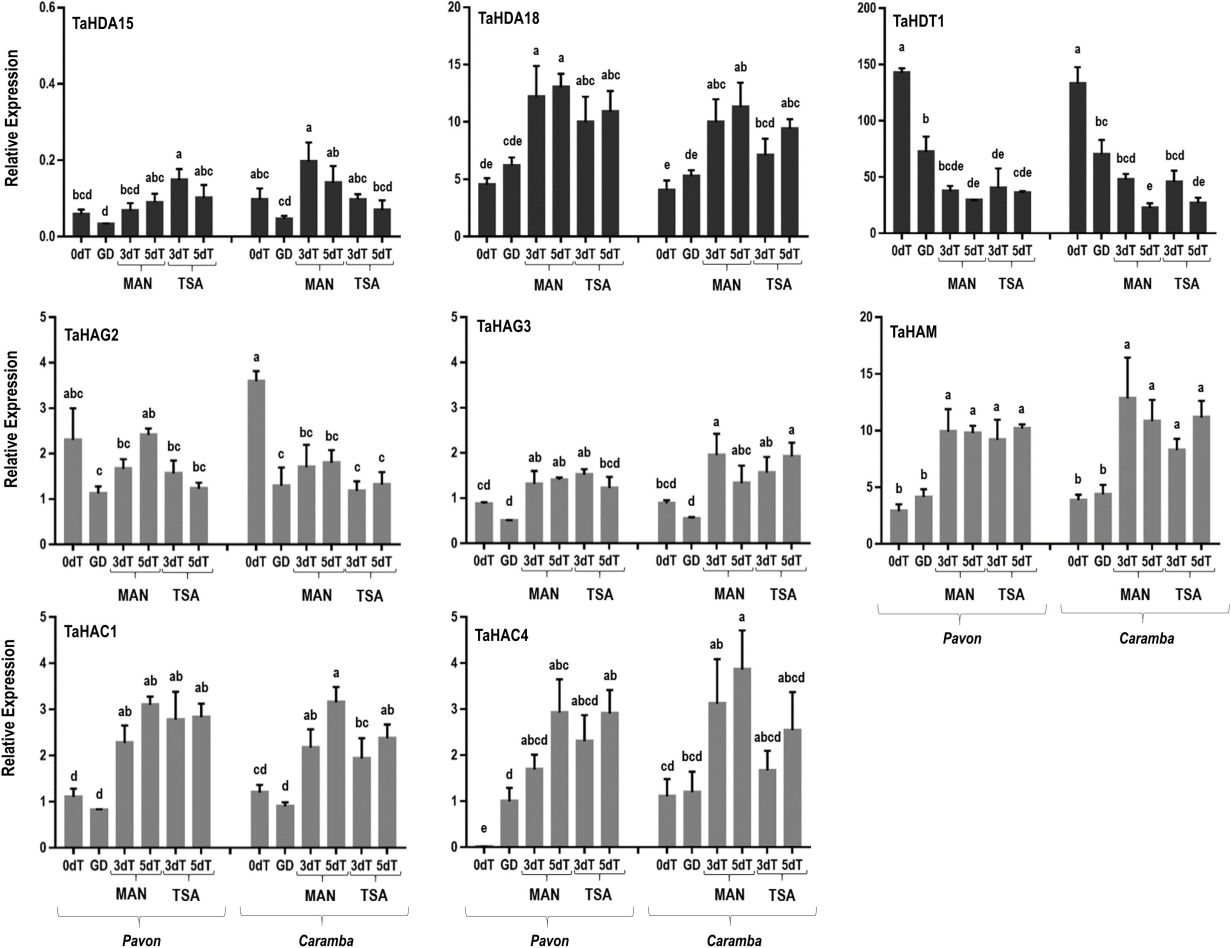
qRT-PCR analysis of histone deacetylases genes (*TaHDA15*, *TaHDA18*, *TaHDT1*, *TaHDT2*, *TaTAF14*) and histone acetyltransferases genes (*TaHAG2*, *TaHAG3*, *TaHAM*, *TaHAC1*, *TaHAC4*) in microspore before stress treatment (0dT), microspores under gametophytic development (GD), microspores after 3 and 5 days with mannitol treatment (3dT-MAN, 5dT-MAN) and after 3 and 5 days with mannitol plus TSA treatment (3dT-TSA, 5dT-TSA) from Pavon (on the left) and Caramba (on the right). Data were normalized using *Ta.27771* and *Ta.2291* as endogenous control genes. Bars represent the standard errors of the means. Relative expression values with the same letter are not significantly different (P<0.05) according to the Duncan test.

In microspores at 0dT, only two HATs genes (*TaHAC4* and *TaHAG2*) showed differential expression between the two cultivars. Both genes were expressed at a lower level in Pavon, although the differences were only significant in *TaHAC4* (**Figure 4**). Also noteworthy was the high expression level of *TaHDT1* in both cultivars. In microspores at GD, *TaHDT1* was down-regulated in both cultivars, *TaHAG2* in Caramba, and *TaHAC4* was up-regulated in Pavon (**Figure 4**).

MAN treatment modified the expression of most HDAC and HAT genes (**Figure 4**). The decrease in *TaHDT1*, compared to 0dT, was even higher than at GD, reaching the lowest values at 5dT in both cultivars. A similar decrease in *TaHAG2* throughout MAN treatment was observed in Caramba, whereas this gene was only slightly down-regulated at 3dT in Pavon. The *TaHDA18* and four HAT genes (*TaHAG3*, *TaHAM*, *TaHAC1* and *TaHAC4)* were up-regulated at 3dT in both cultivars. The expression at 3dT and 5dT in most of the above mentioned genes were similar in both cultivars. *TaHDA15* was not induced in any cultivar, but Pavon showed a lower expression level than Caramba at 3dT (**Figure 4**).

TSA showed a tendency to up-regulate most genes in Pavon and to down-regulate them in Caramba, compared to MAN (**Figure 4**). Notably, TSA treatment caused an increase in *TaHDA15* expression at 3dT in Pavon. In addition, Pavon showed a lower *TaHAG3* expression than Caramba at 5dT-TSA.

The transcriptome and RNA-Seq data set available on the expVIP Wheat Expression Browser was used to analyze the expression of the selected genes under different stresses and in different wheat tissues (**Supplementary Figure 2**). *TaHDA15* and *TaHAG2* were specifically expressed in the spike, whereas a low expression of *TaHAC1* and *TaHAC4* was observed in all tissues. In the RNA-Seq data of bread wheat cv Svilena microspores induced to ME by cold, only *TaHAG3* and *TaHAM* were up-regulated after stress (**Supplementary Figure 3**).

#### Expression of candidate genes involved in microspore embryogenesis induction

Candidate genes involved in key processes associated with ME induction, such as histone methylation, DNA silencing, nuclear fusion, pollen wall development, autophagy, ABA signaling, auxin synthesis, and stress tolerance were also examined.

**Figure 5:**
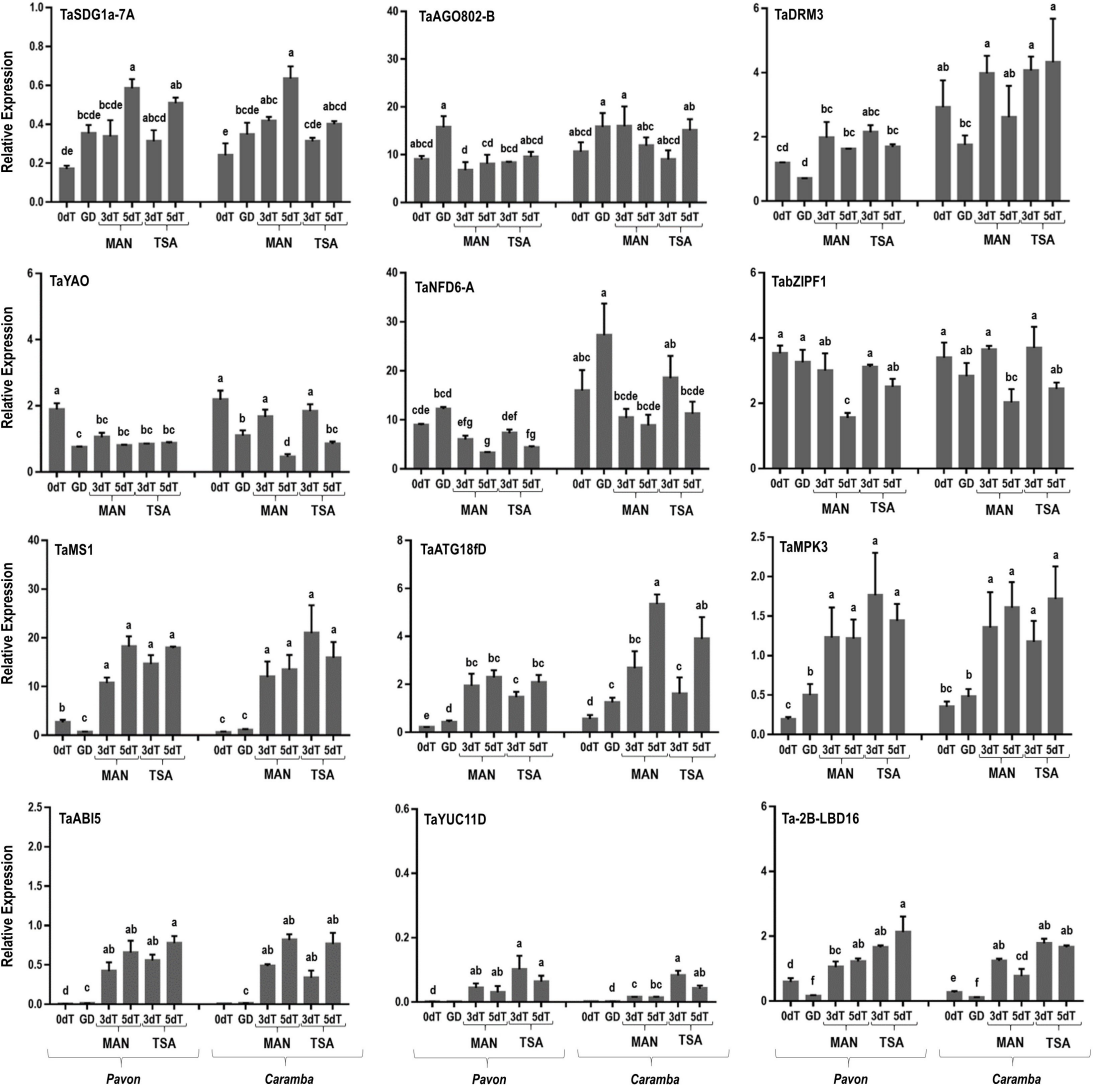
qRT-PCR analysis of key genes involved in microspore embryogenesis induction. Microspores before stress treatment (0dT), microspores under gametophytic development (GD), microspores after 3 and 5 days with mannitol treatment (3dT-MAN, 5dT-MAN) and after 3 and 5 days with mannitol plus TSA treatment (3dT-TSA, 5dT-TSA) from Pavon (on the left) and Caramba (on the right). Data were normalized using *Ta.27771* and *Ta.2291* as endogenous genes control. Bars represent the standard errors of the means. Relative expression values with the same letter are not significantly different (P˂0.05) according to the Duncan test.

In microspores at 0dT, four genes showed differential expression between cultivars: *TaDRM3* (*DOMAINS REARRANGED METHYLTRANSFERASE 3*), encoding a DNA methyltransferase; *TaMS1* (*MALE STERILITY 1*), that encodes a protein required for exine development; *TaATG18fD* (*AUTOPHAGY-ASSOCIATED 18fD*); and *Ta-2B-LBD16* (*LATERAL ORGAN BOUNDARIES DOMAIN 16*), encoding a plant-specific transcription factor (**Figure 5**). Pavon showed higher expression of *TaMS1* and *Ta-2B-LBD16* and lower expression of *TaDRM3* and *TaATG18fD* than Caramba (**Figure 5**). Two genes *TaABI5* (*ABCISIC ACID-INSENSITIVE 5*) and *TaYUC11D* (*INDOLE-3-PYRUVATE MONOOXYGENASE YUCCA 11D*), that encode a basic leucine zipper transcription factor and an auxin synthesis limiting enzyme, respectively, were also differentially expressed between cultivars at a very low level. *TaNFD6-A* (*NUCLEAR FUSION DEFECTIVE 6-A*), encoding a mitochondrial ribosomal protein involved in nuclear membrane fusion, showed a slightly down-regulation in Pavon.

Changes in the expression of several genes were observed when comparing GD with 0dT (**Figure 5**). A reduction in *TaMS1* expression was shown in Pavon. *TaYAO* (*YAOZHE*), that encodes a protein involved in rRNA processing, and *Ta-2B-*LBD16 were down-regulated in both cultivars. In contrast, up-regulation of *TaMPK3* (*MITOGEN-ACTIVATED PROTEIN KINASE 3*) in Pavon and *TaATG18fD* in both cultivars was observed. Genes *TaDRM3*, *TaYAO*, *TaNFD6-A*, *TaATG18fD* and *TaYUC11D* were differentially expressed between cultivars, with Pavon showing a lower level.

MAN treatment at 3dT increased the expression of most genes (*TaMS1*, *TaATG18fD, TaMPK3, TaABI5, TaYUC11D,* and *Ta-2B-LBD16*) compared to 0dT in both cultivars (**Figure 5**). Interestingly, *TaSDG1a-7A* (*SET DOMAIN GENE 1a-7A*), encoding a histone methyltransferase, was up-regulated only in Caramba and *TaYAO* was the only gene down-regulated in Pavon. Differences between cultivars were also observed in *TaAGO802-B, TaDRM3, TaYAO,* and *TaYUC11D*, being *TaYUC11D* the only one with a higher expression in Pavon. When MAN treatment was extended from 3 to 5 days, some genes changed their expression. *TaSDG1a-7A* increased in Pavon, and *TaATG18fD* in Caramba. Also, *TaYAO*, *TabZIPF1* (*BASIC LEUCINE ZIPPER F1*), described as an *E2F-RELATED* (*E2F*) transcription factor, and *Ta-2B-LBD16* were down-regulated in Caramba. Differences between cultivars were also noticed at 5dT-MAN, with *Ta-2B-*LBD16 showing higher expression, and *TaNFD6-A* and *TaATG18fD* lower in Pavon.

TSA only resulted in small changes at 3dT compared to MAN in Caramba. A higher expression of *TaYUC11D* was accompanied by a slightly lower expression of *TaAGO802-B*. Furthermore, *TabZIPF1* in Pavon and *TaYAO* and *Ta-2B-LBD16* in Caramba were up-regulated at 5dT-TSA (**Figure 5**).

The analysis of the transcriptome and RNA-Seq data set available showed that all genes had a low expression level, except *TaAGO802-B*, *TabZIPF1*, *TaMPK3* and *Ta-2B-LBD16* (**Supplementary Figure 2**). Some of these genes were associated with stress response, such as *TaMPK3* and *Ta-2B-LBD16*, while *TaABI5* and *TaNFD6-*A were specifically expressed in grains and roots, respectively, and *TaAGO802-B* and *TaMS1* in spikes. Differences in the expression between homologues of *TaMS1*, *TabZIPF1* and *TaMPK3* were also detected. In the RNA-Seq data of ME induction, few of the studied genes were expressed. *TaAGO802-B* was shown as a constitutive gene, whereas *TaYAO* was mainly expressed in embryogenic microspores, *TaATG18fD* in cold treated and embryogenic microspores, and *TabZIPF1* in fresh and cold treated microspores (**Supplementary Figure 3**).

#### Gene expression correlations and their association with ME induction

Pearson correlation coefficients were calculated to examine the association between the relative expression of genes determining histone acetylation status and genes involved in processes related to ME induction. The data from all samples (0dT, GD, 3dT-MAN, 5dT-MAN, 3dT-TSA, and 5dT-TSA) in the two cultivars were used.

**Figure 6:**
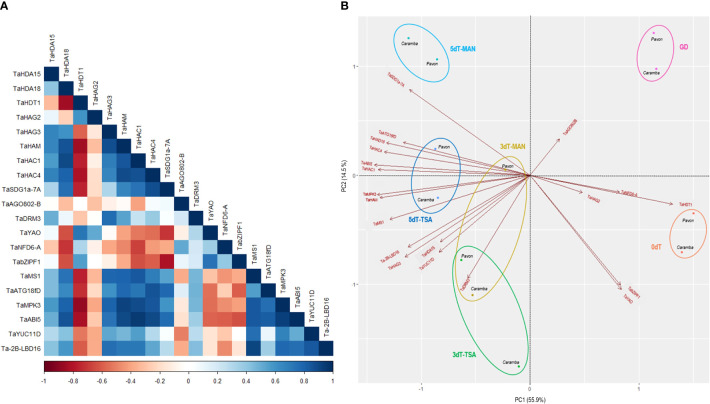
**(A)** Pearson correlation coefficients of histone deacetylases, histone acetyltransferases and key genes involved in ME induction, considering all samples of the two cultivars. **(B)** Principal component analysis (PCA) plot of the 20 genes in Pavon and Caramba. Microspores before stress treatment (0dT), microspores under gametophytic development (GD), microspores after3 and 5 days with mannitol treatment (3dT-MAN, 5dT-MAN) and after 3 and 5 days with mannitol plus TSA treatment (3dT-TSA, 5dT-TSA).

Two groups could be clearly distinguished in the correlation matrix (**Figure 6A**). The first revealed a strong positive correlation between the histone deacetylase *TaHDT1* gene and the set of genes *TaYAO*, *TaNFD6-A* and *TabZIPF1*, and a negative correlation with most of the other genes. *TaHAG2* acetyltransferase was positively correlated with *TaHDT1* and *TaYAO* but was independent of *TaDRM3*, *TaNFD6-A* and *TabZIPF1.* The majority of HDACs and HATs (*TaHDA15* and *TaHDA18*, *TaHAG3*, *TaHAM*, *TaHAC1* and *TaHAC4)* were in the second group that was positively associated with *TaSDG1a-7A*, *TaMS1*, *TaATG18fD*, *TaMPK3*, *TaABI5*, *TaYUC11D* and *Ta-2B-LBD16* (**Figure 6A**).

Principal component analysis (PCA) was performed to identify the genes associated with wheat ME induction. The data set included the relative expression of the genes in all samples from Pavon and Caramba (**Figure 6B**). The two components analysis accounted for 70.4% of the total variance, 55.9% explained by PC1 and 14.5% by PC2. PC1 was mainly determined by *TaHDA18*, *TaHDT1*, *TaHAM*, *TaHAC1*, *TaHAC4*, *TaMS1*, *TaMPK3* and *TaABI5* genes, and PC2 by TaHDA15, *TaHAG3*, *TaSDG1a-7A*, *TaDRM3*, *TaYAO*, *TabZIPF1*, *TaYUC11D* and *Ta-2B-LBD16* genes.

PC1 differentiated untreated (0dT) and GD from MAN and TSA microspores of both cultivars, on the right and left respectively (**Figure 6B**). PC1 showed that *TaHDT1* was strongly associated with microspores at 0dT and GD. *TaYAO* and *TabZIPF1* contributed to the differentiation between 0dT and GD microspores. On the opposite position in PC1, *TaHDA18, TaHAM*, *TaHAC1*, and *TaHAC4* were positively associated with stress treated microspores, accompanied by *TaMS1*, *TaMPK3*, *TaABI5.* The differences between 3dT and 5dT were determined by a strong contribution of *TaSDG1a-7A*, *TaHDA15*, *TaHAG3*, *TaDRM3*, *TaYUC11D and Ta-2B-LBD16*.

## Discussion

Increasing evidence indicates that epigenetic changes are involved in the regulation of biological processes including stress response and developmental reprogramming, both implicated in ME induction (for review see Lloyd and Lister, 2022). Histone PTMs, mainly acetylation and methylation, and DNA methylation have been directly associated with ME induction. Our previous report showed that the simultaneous application of mannitol and TSA increased ME efficiency compared to mannitol in two bread wheat cultivars with different ME response (Castillo et al., 2020). However, it remains a challenge to ascertain the mechanisms by which chromatin organization responds to transcriptional requirements and how TSA modifies them during ME induction.

### Microspores from cultivars with different ME response have a distinctive epigenetic state before induction

Pollen development is characterized by specific chromatin changes, including the dynamics of H3 variants, the progressive loss of active histone marks and the accumulation of repressive marks (Borg and Berger, 2015). Furthermore, transcriptome complexity is significantly reduced after pollen mitosis I (PMI) (Liu et al., 2018a). However, uninucleate microspores (0dT) become transcriptionally active just before PMI, initiating the transition from sporophyte to gametophyte (Nelms and Walbot, 2022).

Histone variants contribute to define different chromatin states (for a review see Probst et al., 2020). The replicative histone H3.1 variant is associated with gene silencing in heterochromatin and the replacement H3.3 variant with active transcription and euchromatin regions (for a review see Probst et al., 2020). The H3.2 identified in wheat belongs to the histone H3.3 class (Waterborg, 1991). According to Otero et al. (2016), the ratio between H3.1 and H3.3 could be related to a proliferative state of the cell in *Arabidopsis*. Interestingly, the H3.1/H3.2 ratio was higher in Pavon than in Caramba at 0dT, that could indicate an elevated cell division potential of Pavon (Otero et al., 2016). Differences in H3.1 acetylation have been associated with diverse heterochromatin domains and compaction levels (Jasencakova et al., 2001). The lower H3.1 acetylation suggests a higher heterochromatin compaction in Pavon (**Figure 1**).

One of the major epigenetic differences between cultivars at 0dT was the higher percentages of acetylated microspores in Pavon, highlighting the percentage of H3K9ac (**Figure 3A**). At GD, the percentage of labeled microspores with H3K9ac decreased in Pavon whereas this mark was absent in Caramba (**Figure 3A**). It is known that H3K9ac mark is present in microspores but is removed during pollen development (Pandey et al., 2013; Liu et al., 2018a). In accordance, Pavon microspores could be at a less advanced stage in pollen development. Curiously, H4K5ac was localized in the cytoplasm, including the vacuole, in both cultivars at GD (**Figure 2**), the increase of labeled microspores with this mark in Caramba being noteworthy (**Figure 3A**). The presence of H4K5ac in the cytoplasm has been previously described in barley and *Brachypodium* (Braszewska-Zalewska et al., 2013; Wolny et al., 2014). These authors propose that newly synthetized acetylated H4, prior to DNA replication, is present in the cytoplasm while the recycling of acetylated H4 takes place in the vacuole. Differences in H4K5ac dynamics suggests that Pavon is less primed to enter in DNA replication. Histone acetylation depends on the balance between HDAC and HAT. No major differences in *HDAC* or *HAT* expression were observed between cultivars at 0dT, except for *TaHAC4* that showed a lower level in Pavon (**Figure 4**). *TaHAC4* has been associated with the enrichment of H3K9ac in *Arabidopsis* (Li et al., 2022). In contrast, the expression level of this gene was negatively correlated with the percentage of microspores with H3K9ac in both cultivars.

In *Arabidopsis*, the diploid to haploid transition is governed by the loss of H3K9me2 mark, and the transition back to diploid by the loss of H3K27me3 (Borg et al., 2021). However, H3K9me2 mark persisted throughout pollen development in rye and barley (Houben et al., 2011; Pandey et al., 2013). In this study, H3K9me2 was absent in Pavon and present in Caramba at 0dT, and the opposite was observed at GD (**Figure 2**). This could be associated with small adjustments to control transposon reactivation in the two cultivars during pollen development (Pecinka et al., 2010). Interestingly, in Caramba, H3K27me3 was exclusively in the cytoplasm that could also be related to an accumulation of histones prior to DNA replication as indicated in barley (Pandey et al., 2017). However, the progressive decrease of the percentage of labeled nuclei and the enrichment of H3K27me3 in the cytoplasm reinforce the less advanced stage in pollen development of Pavon microspores.

Differences in the epigenetic state of the cultivars at 0dT were barely reflected at the transcriptional level of the genes involved in ME induction, with the exception of *TaDRM3* and *TaNFD6-A*. *DRM3* participates in *de novo* DNA methylation *via* RNA (RdDM) and is crucial to maintain CHH methylation, although the levels in microspores are low (Henderson et al., 2010; Gehring, 2019). *TaNFD6-A* encodes a protein related to nuclear membrane fusion, playing an important role in female and male gametogenesis (Portereiko et al., 2006). At 0dT, Caramba microspores had a higher expression of both genes, but *TaDRM3* decreased and *TaNFD6-A* increased in both cultivars at GD. Other genes related to pollen development, as *TaMS1* and *Ta-2B-LBD16* (Li et al., 2021; Xu et al., 2021), had a higher expression at 0dT in Pavon and were down-regulated at GD in both cultivars. The expression pattern of these genes confirmed that Pavon microspores were more delayed in pollen development.

### MAN and TSA treatments modified the epigenetic state of microspores initiating a new cell fate

The relationship between epigenetic modifications, transcriptional changes and ME induction is largely unknown. The analysis of epigenetic dynamics in coordination with gene regulation offers new opportunities to study the mechanisms underlying microspore reprogramming towards embryogenesis.

#### MAN and TSA treatments modified the H3 variants ratio and H3 and H4 acetylation

H3 variants are involved in transcriptional regulation in response to stress (Yung et al., 2021). However, no previous information about the role of histone variants in ME induction is available. In this study, MAN and TSA raised the H3.1/H3.2 ratio in both cultivars, highlighting the TSA effect at 3dT (**Figure 1**). These results agreed with a high cell division potential (Otero et al., 2016), and with the higher number of pro-embryos produced by TSA (Castillo et al., 2020). Histone hyperacetylation processes were observed in MAN and TSA (**Figure 1**), accordingly with previous results reported in *Brassica* ME induction by heat and TSA (Li et al., 2014a). The results obtained in this study confirm the higher hyperacetylation capacity of the H3.2 variant described by Waterborg (1991). The effect of TSA on H3.2 hyperacetylation enhancement at 3dT (**Figure 1**) could be an important factor for ME induction in both cultivars. MAN and TSA caused only minor H4 deacetylation/acetylation processes, although H4 acetylation was slightly higher with TSA in both cultivars at 3dT.

#### The dynamics of PTMs in response to MAN and TSA changed microspore epigenetic state

Long-duration and medium-intensity stress treatments have epigenetic characteristics that allow for a greater adaptation and survival (Pecinka et al., 2010). Thus, acetylation is positively associated with the activation of stress response genes in plants (Kong et al, 2020), but deacetylation is also essential to cause growth responses to warm temperature (Tasset et al., 2018).

In this study, the effect of the MAN and TSA on histone acetylation depended on the epigenetic state of the microspores at 0dT. A strong decrease in the percentage of microspores with H3K9ac in Pavon and a large increase in H3K9ac and H4K16ac in Caramba were observed at 3dT (**Figure 3A**). Interestingly, both PTMs were detected in the nuclei after treatments in this study, whereas H3K9ac was mainly located in the cytoplasm in barley (Pandey et al., 2017). The cytoplasmic H4K5ac did prove to be distinctive between treatments in Caramba, emerging at 3dT with TSA but at 5dT with MAN (**Figure 2B**). As mentioned above, the presence of H4K5ac in the cytoplasm could point out to a nascent acetylated H4 for DNA synthesis. Therefore, TSA could accelerate the replication processes in the mid-low responding cultivar. Curiously, one effect of TSA in maize seedlings is the hyperacetylation of H4K5, which is associated with cell cycle arrest in prophase (Wang et al., 2016). Furthermore, DNA replication arrested in G1 is related to the accumulation of the cytoplasmic H4K5ac signals in dry seeds of *Brachypodium* (Wolny et al., 2014).

The percentages of labeled microspores with H3K9me2 and H3K27me3 silencing marks during treatments seemed to be determined by the cultivar and the epigenetic state of the microspore at 0dT, similarly to histone acetylation. In MAN and TSA, H3K9me2 emerged at 3dT in Pavon but it was present in Caramba at 0dT and throughout treatments. A striking increase in the percentage of labeled microspores at 5dT-TSA was observed in Caramba (**Figure 3B**). However, the presence of H3K9me2 was noticed during ME induction in a high responding barley cultivar (Pandey et al., 2017). The loss of H3K27me3 accompanies callus induction and plant regeneration in *Arabidopsis* (He et al., 2012; Yan et al., 2020). In this study, the presence of this mark in Pavon nuclei could indicate some level of silencing, which is not contradictory to an active response to ME induction. In Caramba, H3K27me3 is restrictive to the cytoplasm as in a barley high responding cultivar to ME (Pandey et al., 2017). Even if there were differences between cultivars, it was not possible to establish a direct correlation between methylation marks and ME induced by MAN and TSA.

#### MAN and TSA caused transcriptional changes for the elimination of gametophytic pathway and the initiation of a new developmental program

The gene networks regulated by histone PTMs are still poorly characterized (Kumar et al., 2021). In addition, few reports are focused on transcriptional changes accompanying wheat ME induction (Seifert et al., 2016; Jensen, 2021), and the direct impact of PTMs dynamics on key genes related to ME is unknown.

After 3 days of MAN or TSA treatments, most genes studied were up-regulated compared to 0dT and GD. Only a few genes associated with 0dT or GD in PCA analysis, *TaHDT1, TaHAG2, TaYAO*, *TaNFD6-A*, *TabZIPF1* and *TaAGO802-B* were down-regulated (**Figures 4**, **5**, **6**). *TaYAO*, *TaNFD6-A*, *TabZIPF1* and *TaAGO802* were directly related to different pollen development processes as 18S pre-rRNA processing, zinc homeostasis, DNA silencing, or other unknown functions (Portereiko et al., 2006; Li et al., 2010; Evens et al., 2017; Akond et al., 2020). Disruption of the gametophytic developmental pathway has been reported as a key process in barley ME induction with mannitol (Muñoz-Amatriaín et al., 2006; Muñoz-Amatriaín et al., 2009). In this study, a progressive down-regulation of most genes associated with the disruption of the gametophytic development was observed, with a tendency to be faster in Pavon. Surprisingly, TSA caused a slower down-regulation of *TaYAO* and *TaNFD6-A* in Caramba, and *TabZIPF1* in Pavon. *TaAGO802-B* deserves an especial mention, since it exhibited a remarkable difference between MAN and TSA, with opposite expression in Caramba at 3dT (**Figure 5**).

The epigenetic control of the transcriptional regulation of these genes could be associated with *TaHDT1* and *TaHAG2. TaHDT1* sharply decreased at 3dT in both treatments and cultivars as compared to 0dT. This gene is a negative regulator of biotic defense response in wheat (Zhi et al., 2020). Accordingly, the activation of pathogen defense genes has been previously described in barley and wheat ME induction (Muñoz-Amatriaín et al., 2006; Soriano, 2008). Moreover, *HDT1* binds to the *YAO* promoter during rRNA processing (Luo et al., 2022). Therefore, the down-regulation of *TaHDT1* could lead to a reduction on rRNA processing, allowing the elimination of gametophytic development by prioritizing the synthesis of stress-response RNAs. *TaHAG2* is activated in response to drought and medium temperature stresses (Gao et al., 2021; Li et al., 2022). In this study, *TaHAG2* was down-regulated with the stress, showing only slight differences between treatments.

*TaHDA15*, *TaHAG3*, *TaHAM, TaYUC11D*, *Ta-2B-LBD16, TaMS1* and *TaDRM3* constitute a network according to a PCA analysis (**Figure 6**), characterized by activation a 3dT and few expression changes at 5dT (**Figures 4**, **5**). *TaYUC11D* is a limiting enzyme in auxin synthesis (Yang et al., 2021). In ME, auxin accumulation is localized in uninucleate microspores after induction in *Brassica* (Dubas et al., 2014; Soriano et al., 2014). *De novo* auxin biosynthesis is noticed at early multicellular embryo in barley and *Brassica* ME, increasing during embryo development. (Rodríguez-Sanz et al., 2014; Pérez-Pérez et al., 2019). In addition, it has been suggested that *YUC11* is responsible of auxin biosynthesis require for zygotic embryo polarization in *Arabidopsis* (Larsson et al., 2017). Furthermore, *YUCCA* genes also play a crucial role in morphology changes associated with survival in a medium-long temperature stress (Casal and Balasubramanian, 2019). Interestingly, TSA increased the expression of *TaYUC11D*, particularly in the mid-low responding cultivar Caramba (**Figure 5**). These results agree with the up-regulation of auxin-related genes produced by TSA in *Brassica* ME (Li et al., 2014a). Moreover, TSA also caused an up-regulation of the auxin related gene *Ta-2B-LBD16*, in Caramba at 5dT. *Ta-2B-LBD16* is an orthologist of *AtLBD40* that promotes auxin signaling-dependent lateral organ development (Zentella et al., 2007; Okushima et al., 2007). TSA has also been associated with an up-regulation of genes encoding cell wall mobilization enzymes in *Brassica* ME (Li et al., 2014a). In this study, *TaMS1*, which is required for pollen exine development (Liu et al., 2018b), was up-regulated not only by TSA but also by MAN. A special mention of *TaDRM3*, a DNA silencing component, should also be made since it was up-regulated by treatments, maintaining the differences between cultivars previously observed at 0dT.

Diverse histone acetylation regulatory genes are included in this network, but only *TaHAG3* and *TaHAM* showed slight differences between treatments in Caramba, highlighting *HAG3* expression at 5dT-TSA (**Figure 4**). Interestingly, orthologists of *TaHDA15*, *TaHAG3* and *TaHAM* have been described to promote hypocotyl elongation by up-regulation of auxin response and cell wall modifications genes in darkness in *Arabidopsis* (Tang et al., 2017; Woloszynska et al., 2018; Peng et al., 2018). Therefore, histone acetylation could be directly involved in the up-regulation of auxin related genes in this network.

The last network was characterized by the highest expression levels at 5dT, including *TaABI5*, *TaATG18fD, TaSDG1a-7A*, *TaHDA18, TaHAC1* and *TaHAC4* (**Figure 6**). The role of ABA in ME induction is unclear (for review see Żur et al., 2015). It has been proposed that each genotype requires a basal level of ABA to initiate a signaling cascade for ME (Żur et al., 2012). However, *TaABI5* was expressed at low levels at 0dT, and slightly increased throughout the treatments at a similar rate in the two cultivars. This could be in line with the requirement of the novo ABA synthesis to induce barley ME reported by Hoekstra et al. (1996). The up-regulation of *BnATG8*, accompanied by an accumulation of autophagosomes, has been previously described in *Brassica* ME (Berenguer et al., 2020). In this study, the mid-low responding cultivar had a higher expression of *TaATG18fD*, suggesting a greater cellular component recycling in stress response. Finally, the gene *TaSDG1a-7A*, an orthologist of AtCLF (*CURLY LEAF*) (Strejčková et al., 2020) that is a PRC2 component (polycomb repressive complex 2), was up-regulated particularly at 5dT-MAN. The CLF/H3K27me3 complex could play a role in controlling the transcription of developmental genes in stress-response (Avramova, 2015). The up-regulation of *CLF* has been previously associated with ME induction by mannitol in barley (Muñoz-Amatriaín et al., 2009), and by cold in perennial ryegrass (Begheyn et al., 2018).

The *TaHDA18, TaHAC1* and *TaHAC4* genes could be implicated in the epigenetic control of the network. It has been reported that they are related to the regulation of developmental processes mediated by hormone signaling, including ABA, ethylene or auxins (Xu et al., 2005; Li et al., 2014b; Li et al., 2022). These genes showed a slightly higher expression with MAN than TSA, especially in Caramba.

In this report, for the first time, a comprehensive analysis of the epigenetic dynamics involved in ME induction has been performed applying two treatments of different efficiency, in two bread wheat cultivars with different response. The epigenetic state of the microspores before induction was distinctive between cultivars. Evidences obtained in this study allow us to conclude that microspores of the high responding cultivar at the onset of ME induction were at a less advanced stage in pollen development and had a higher cell division potential. The initial differences determined the dynamics of histone PTMs to MAN and TSA. A response pattern could be established for the percentages of acetylation but not for methylation, during treatments. MAN and TSA increased the H3.1/H3.2 ratio and the hyperacetylation of H3. Moreover, there was a down-regulation of pollen developmental genes and an up-regulation of genes related to stress and hormonal response and morphological changes. The effect of TSA pointed out the most significant processes for wheat ME induction. In the mid-low responding cultivar, TSA enhanced histone hyperacetylation and cell division potential, and promoted an early initiation of DNA replication mechanisms. In addition, the down-regulation of pollen development genes was slowed down whereas the expression of genes associated with morphological changes, mainly mediated by auxin signaling, was increased. This study answers key questions about the role of epigenetic modifications and their relationship with transcriptional requirements in wheat ME induction, and it is a starting point for new studies and approaches to improve ME efficiency in a wide range of wheat genotypes and other crops.

## Data availability statement

The original contributions presented in the study are included in the article/**Supplementary Materials**. Further inquiries can be directed to the corresponding author.

## Author contributions

Conceptualization AMC and MPV; methodology IV-R, AMC, MAB, and MPV; microspore and RNA sampling IV-R, AMC, and MPV; immunofluorescence analysis IV-R, AMC, and MAB; gene expression analysis IV-R and MPV; writing-original draft preparation IV-R, AMC, and MPV; writing-review and editing IV-R, AMC, MAB, and MPV; supervisor, project administration and funding acquisition AMC and MPV. All authors contributed to the article and approved the submitted version.
