# Supplementary material for: Microspore embryogenesis induction by mannitol and TSA results in a complex regulation of epigenetic dynamics and gene expression in bread wheat
Source: Front Plant Sci. 2023 Jan 9;13:1058421. doi: 10.3389/fpls.2022.1058421 (PMC9868772; doi:10.3389/fpls.2022.1058421)
Supplement: Supplementary file 1 [file DataSheet_1.docx]

**Supplementary Table 1**. *HDACs*, *HATs* and genes involved in wheat ME induction were selected based on an Affymetrix Barley1 GeneChip analysis (Sánchez-Díaz, 2014). The corresponding orthologues in *Arabidopsis thaliana* and *Oryza sativa* Japonica Group are included.

| **Barley probe set** | **Gene name** | **Gene ID (RefSeqv1)** | ***Arabidopsis thaliana*** | ***Oryza sativa*** |
| --- | --- | --- | --- | --- |
| Contig13185_at | *TaHDA15* | TraesCS5A02G065300 | HDA15 (AT3G18520) | OsHDA704 (Os07g0164100) |
|  |  | TraesCS5B02G072100 |  |  |
|  |  | TraesCS5D02G076100 |  |  |
| Contig13757_at | *TaHDA18* | TraesCS2A02G177100 | HDA05 (AT5G61060) | OsHDA713 (Os07g0602200) |
|  |  | TraesCS2B02G204100 | HDA18 (AT5G61070) |  |
|  |  | TraesCS2D02G185200 |  |  |
| Contig1625_at | *TaHDT1* | TraesCS1A02G445700 | HDT1 (AT3G44750) | OsHDT1, OsHDT701 (Os05g0597100) |
|  |  | TraesCS1D02G454400 | HDT3 (AT5G03740) |  |
| Contig12304_at | *TaHAG2* | TraesCS5A02G197700 | HAG2 (AT5G56740) | OsHAG704 (Os09g0347800) |
|  |  | TraesCS5B02G186000 |  |  |
|  |  | TraesCS5D02G193200 |  |  |
| Contig17371_at | *TaHAG3* | TraesCS2A02G320900 | HAG3 (AT5G50320) | OsHAG703 (Os04g0484900) |
|  |  | TraesCS2B02G361800 |  |  |
|  |  | TraesCS2D02G341600 |  |  |
| Contig5957_s_at | *TaHAM* | TraesCS2A02G159700 | HAM1 (AT5G64610) | OsHAM701 (Os07g0626600) |
|  |  | TraesCS2B02G185300 | HAM2 (AT5G09740) |  |
|  |  | TraesCS2D02G166900 |  |  |
| HF08A05r_at | *TaHAC1* | TraesCS3A02G524800 | HAC2 (AT1G67220) | OsHAC701 (Os01g0246100) |
|  |  | TraesCS3B02G592100 |  |  |
|  |  | TraesCS3D02G530000 |  |  |
| Contig24596_at | *TaHAC4* | TraesCS7A02G414500 | HAC04 (AT1G55970) | OsHAC704 (Os06g0704800) |
|  |  | TraesCS7B02G314400 | HAC5 (AT3G12980) |  |
|  |  | TraesCS7D02G407600 |  |  |
| Contig24842_at | *TaSDG1a-7A* | TraesCS7A02G128600 | CLF (AT2G23380) | CLF (Os06g0275500) |
|  |  | TraesCS7B02G028500 |  |  |
|  |  | TraesCS7D02G127400 |  |  |
| Contig4579_s_at | *TaAGO802-B* | TraesCS3B02G217300 | AGO4 (AT2G27040) | OsAGO4a (Os01g0275600) |
|  |  |  | AGO9 (AT5G21150) |  |
| Contig11117_at | *TaDRM3* | TraesCS5A02G051400 | DRM3 (AT3G17310) | OsDRM3 (Os05g0133900) |
|  |  | TraesCS5B02G057300 |  |  |
|  |  | TraesCS5D02G062400 |  |  |
| Contig11452_at | *TaYAO* | TraesCS5A02G043100 | YAO (AT4G05410) | OsWD40-78 (Os03g0625900) |
|  |  | TraesCS5D02G051600 |  |  |
| Contig19167_s_at | *TaNFD6-A* | TraesCS2A02G124300 | NFD6 (AT2G20585) | NFD6 (Os01g0754000) |
|  |  | TraesCS2D02G127100 |  |  |
| Contig12955_at | *TabZIPF1* | TraesCS7A02G530300 |  | OsbZIP53 (Os06g0716800) |
|  |  | TraesCS7B02G447900 |  |  |
|  |  | TraesCS7D02G518100 |  |  |
| / | *TaMS1* | TraesCS4A02G295900 |  | EPAD1 (Os03g0663900) |
|  |  | TraesCS4B02G017900 |  |  |
| Contig11426_at | *TaATG18fD* | TraesCS3A02G324900 | ATG18F (AT5G54730) | OsATG18e (Os01g0786900) |
|  |  | TraesCS3B02G353900 |  |  |
|  |  | TraesCS3D02G318200 |  |  |
| Contig5531_at | TaMPK3 | TraesCS4A02G106400 | MPK3(AT3G45640) | OsMSRMK2 (Os03g0285800) |
|  |  | TraesCS4B02G197800 |  |  |
|  |  | TraesCS4D02G198600 |  |  |
| Contig8163_at | *TaABI5* | TraesCS3D02G364900 | ABI5 (AT2G36270) | OsABI5-1 (Os01g0859300) |
| Contig18217_at | *TaYUC11D* | TraesCS5D02G225200 |  | OsYUCCA9 (Os01g0273800) |
|  |  | TraesCS5A02G217200 |  |  |
|  |  | TraesCS5B02G216000 |  |  |
| Contig8459_at | *Ta-2B-LBD16* | TraesCS2B02G289800 | LBD40 (AT1G67100) |  |

**Supplementary Table 2**. Primers used for RT-qPCR analysis.

| **Primer name** | **Sequence** | **Reference** |
| --- | --- | --- |
| *TaHDA15-F* | TGGTCGTGAGAACTGTGGAG | Jin et al., 2020 |
| *TaHDA15-R* | TCTTGGCATGCTGACCACAA |  |
| *TaHDA18-F* | TCCTTTGATTGGCTTGTCCG | Li et al., 2022 |
| *TaHDA18-R* | CTTCCCTTCCACGAGTCCCT |  |
| *TaHDT1-F* | AAGAAGAGGGCGGCTGGAA | Li et al., 2022 |
| *TaHDT1-R* | CTCGCTGTTGAATGTCTTGCTG |  |
| *TaHAG2-F* | ACTTGGTGCTATGGTCTTGTCTCT | Li et al., 2022 |
| *TaHAG2-R* | TTTGGTGCGGTCGTAGATGC |  |
| *TaHAG3-F* | CTGGTTTCAAGGTGGTTGCG | Gao et al., 2021 |
| *TaHAG3-R* | GCAGCTCTGGTGGGTAGTTT |  |
| *TaHAM-F* | GGTAAAGTGGGAACACCGGA | Gao et al., 2021 |
| *TaHAM-R* | TCCAGATCAGCTTGCTCACG |  |
| *TaHAC1-F* | GCTGGCGGTGTGAAATTTGT | Gao et al., 2021 |
| *TaHAC1-R* | TGAATCTCTGCAAGCTCGGG |  |
| *TaHAC4-F* | ATAAATGTCGCACCGCTGGTT | Li et al., 2022 |
| *TaHAC4-R* | TGCTGCGTCCACCTTAGATG |  |
| *TaSDG1a-7A-F* | TGAGAATTCGTCGTTCCTG | Batra et al., 2020 |
| *TaSDG1a-7A-R* | TACCCTGTGATCGCCTGCT |  |
| *TaAGO802B-F* | CCTGTTGGGACTCCTGGTATGTTGC | Singh et al., 2013 |
| *TaAGO802B-R* | AGTAAAGGTTCAACAGCAGCATCCTA |  |
| *TaDRM3-F* | AAACCCACCGGACAGGATTG | This work |
| *TaDRM3-R* | GCAATTTCTGCACCCCCAAC |  |
| *TaYAO-F* | TTCTGATCTGGCTGCGTCTG | This work |
| *TaYAO-R* | TCCAGTGGCAAGTCGAACAA |  |
| *TaNFD6-A-F* | GTCTGGGATGAAGAGACGCC | This work |
| *TaNFD6-A-R* | CAGGCTCCCACAACAAGAGA |  |
| *TabZIPF1-F* | GTCATGAGTGGCCAGGTTTT | Placido et al., 2013 |
| *TabZIPF1-R* | GCCAATAGTCTCTCGCAAGG |  |
| *TaMS1-F* | ACATCATCCTCTGAGTCGCG | Wang et al., 2017 |
| *TaMS1-R* | GACCACGCAAACACGTACG |  |
| *TaATG18fD-F* | GCTACTCCCGGACTTATGCC | This work |
| *TaATG18fD-R* | TTACACACGCACGGGAAAGA |  |
| *TaMPK3 -F* | ATGAGCGAGTCTGACCTGGAGTTC | Yan et al., 2017 |
| *TaMPK3 -R* | AGTAGTGAGCCTGCCGGGTTCTTC |  |
| *TaABI5-F* | CATGATGAACTGCATGGGCG | Zhang et al., 2019 |
| *TaABI5-R* | TCAGCTTTCAGACGAGCGTT |  |
| *TaYUC11D-F* | TCCATGAAGATCCACACCCC | This work |
| *TaYUC11D-R* | TCCCGTCCACAGTCTTCATC |  |
| *Ta-2B-LBD16-F* | GCACATTTACCTTGTGAAAACATAGAG | Wang et al., 2021 |
| *Ta-2B-LBD16-R* | GCTATTGTCTGTACATTGCAACCATG |  |


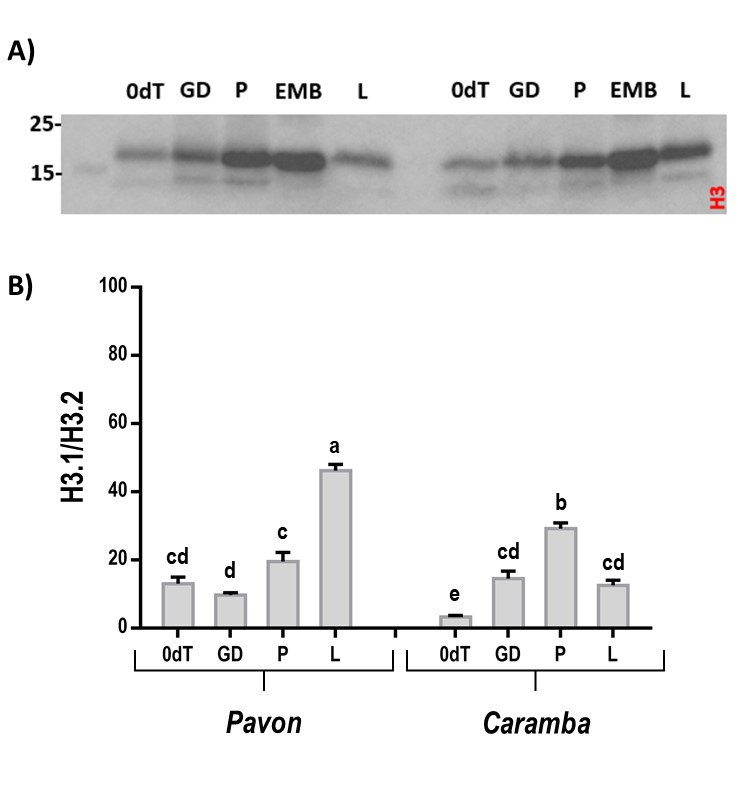


**Supplementary Figure 1**. Analysis of the histone H3 variants (H3.1 and H3.2) in different wheat tissues. A) Immunoblot of H3 in microspores before stress treatment (0dT), microspores under gametophytic development (GD), mature pollen grains (P), zygotic embryos (14 days after pollination, EMB), and leaves (L). B) Quantitative analysis of the H3.1/H3.2 ratio in 0dT, GD, P and L from Pavon (on the left) and Caramba (on the right). Bars represent the standard errors of the means. Histone H3.1/H3.2 ratios with the same letter are not significantly different (P<0.05) according to the Duncan test.


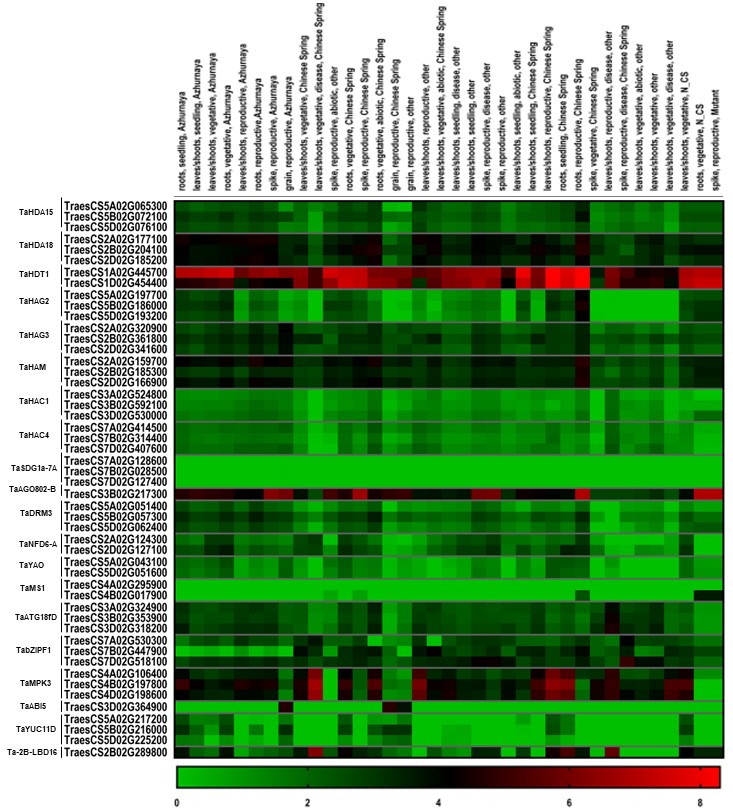


**Supplementary Figure 2.** Expression of the HDAC and HAT genes, and genes involved in key processes of ME induction under different stresses and several tissues of wheat, based on an available transcriptome and RNA-seq data set from expVIP Wheat Expression Browser (http://www.wheat-expression.com). Values are in fragments per kilobase of exon per million reads mapped (FPKM).


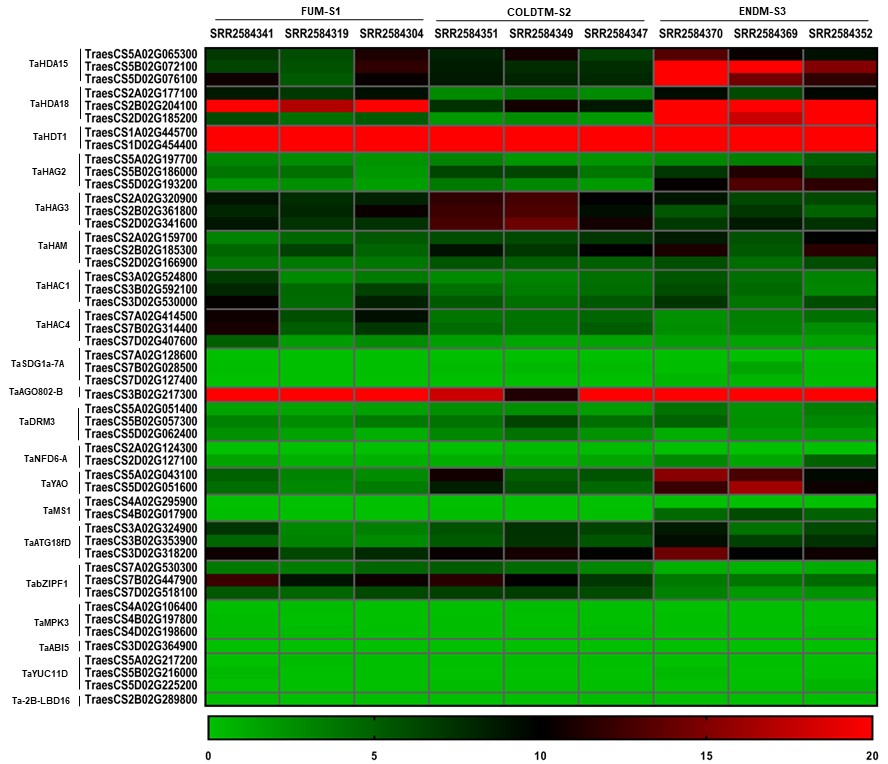


**Supplementary Figure 3**. Expression of the HDAC and HAT genes, and genes involved in key processes of ME induction based on an available RNA-seq data set analysis of ME induced by cold in bread wheat cv. Svilena. Data downloaded from expVIP Wheat Expression Browser (http://www.wheat-expression.com). Values are in fragments per kilobase of exon per million reads mapped (FPKM). FUM-S1: fresh uninucleate microspore- stage1; COLDTM-S2: cold treated microspore-stage2; ENDM-S3: early nuclear division microspore-stage3.

References

Batra, R., Gautam, T., Pal, S., Chaturvedi, D., Jan, I., Balyan, H. S. et al. (2020). Identification and characterization of SET domain family genes in bread wheat (*Triticum aestivum L*.). *Scientific Reports*, 10, 1-14.

Jin, P., Gao, S., He, L., Xu, M., Zhang, T., Zhang, F. et al. (2020). Genome-wide identification and expression analysis of the histone deacetylase gene family in wheat (*Triticum aestivum L*.). *Plants*, 10, 19.

Placido, D. F., Campbell, M. T., Folsom, J. J., Cui, X., Kruger, G. R., Baenziger, P. S. et al. (2013). Introgression of novel traits from a wild wheat relative improves drought adaptation in wheat. *Plant Physiology*, 161, 1806-1819.

Singh, M., Singh, S., Randhawa, H., Singh, J. (2013). Polymorphic homoeolog of key gene of RdDM pathway, ARGONAUTE4_9 class is associated with pre-harvest sprouting in wheat (*Triticum aestivum L*.). *PLOS One*, 8, e77009.

Wang, Z., Li, J., Chen, S., Heng, Y., Chen, Z., Yang, J. et al. (2017). Poaceae-specific MS1 encodes a phospholipid-binding protein for male fertility in bread wheat. *Proceedings of the National Academy of Sciences*, 114, 12614-12619.

Wang, Z., Zhang, R., Cheng, Y., Lei, P., Song, W., Zheng, W. et al. (2021). Genome-Wide Identification, Evolution, and Expression Analysis of LBD Transcription Factor Family in Bread Wheat (*Triticum aestivum L*.). *Frontiers in Plant Sciences* 12:721253

Yan, J., Su, P., Wei, Z., Nevo, E., Kong, L. (2017). Genome-wide identification, classification, evolutionary analysis and gene expression patterns of the protein kinase gene family in wheat and *Aegilops tauschii*. *Plant Molecular Biology*, 95, 227-242.

Zhang, T., Liu, P., Zhong, K., Zhang, F., Xu, M., He, L.et al. (2019). Wheat yellow mosaic virus NIb interacting with host light induced protein (LIP) facilitates its infection through perturbing the abscisic acid pathway in wheat. *Biology*, 8, 80.
